# Supplementary figures and images for: Accumulation of road salt in a calcareous fen: Kampoosa Bog, western Massachusetts
Source: PLoS One. 2024 Oct 31;19(10):e0312259. doi: 10.1371/journal.pone.0312259 (PMC11527221; doi:10.1371/journal.pone.0312259)

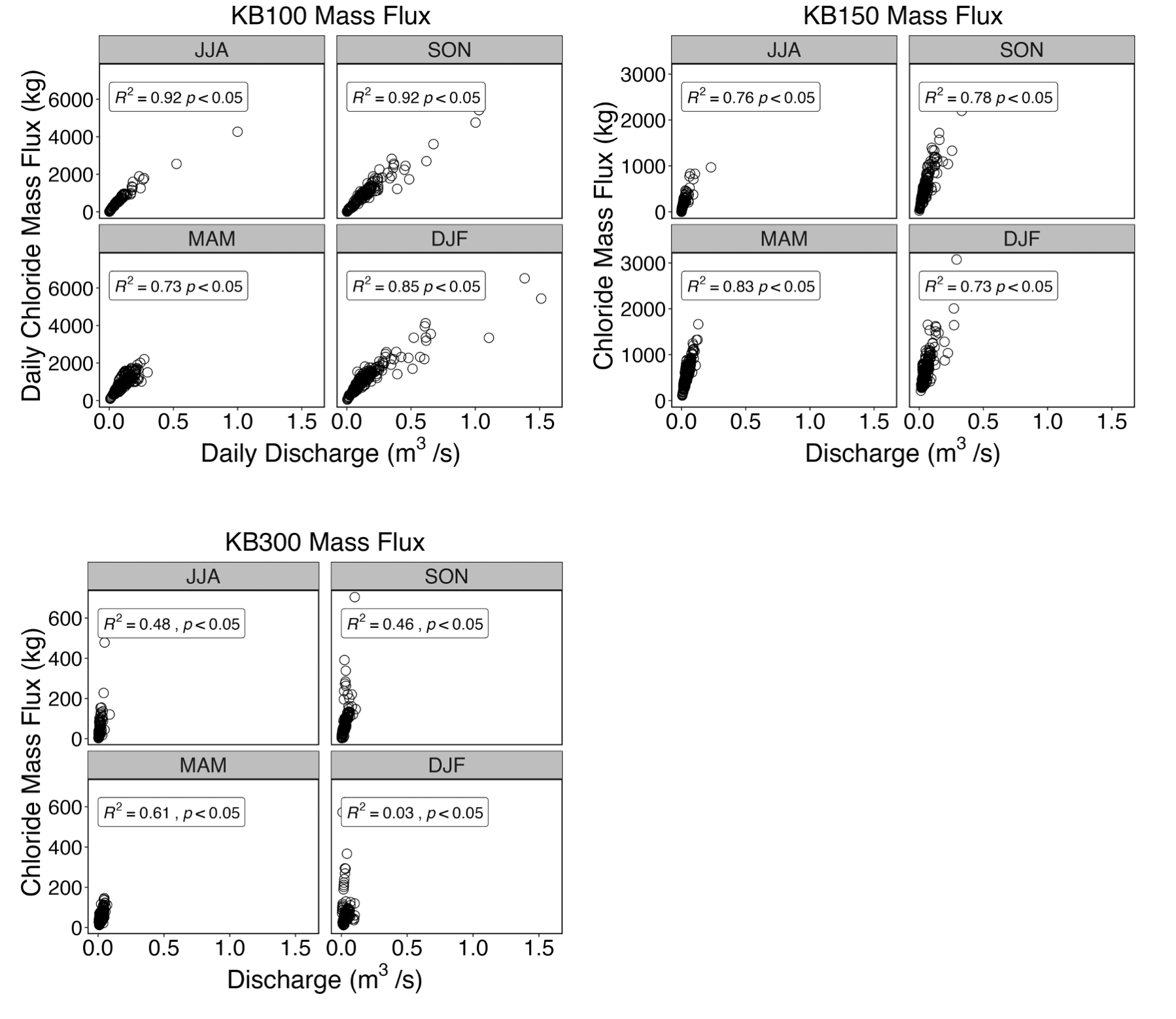

Supplement: S1 Fig — (TIF) [file pone.0312259.s001.tif]

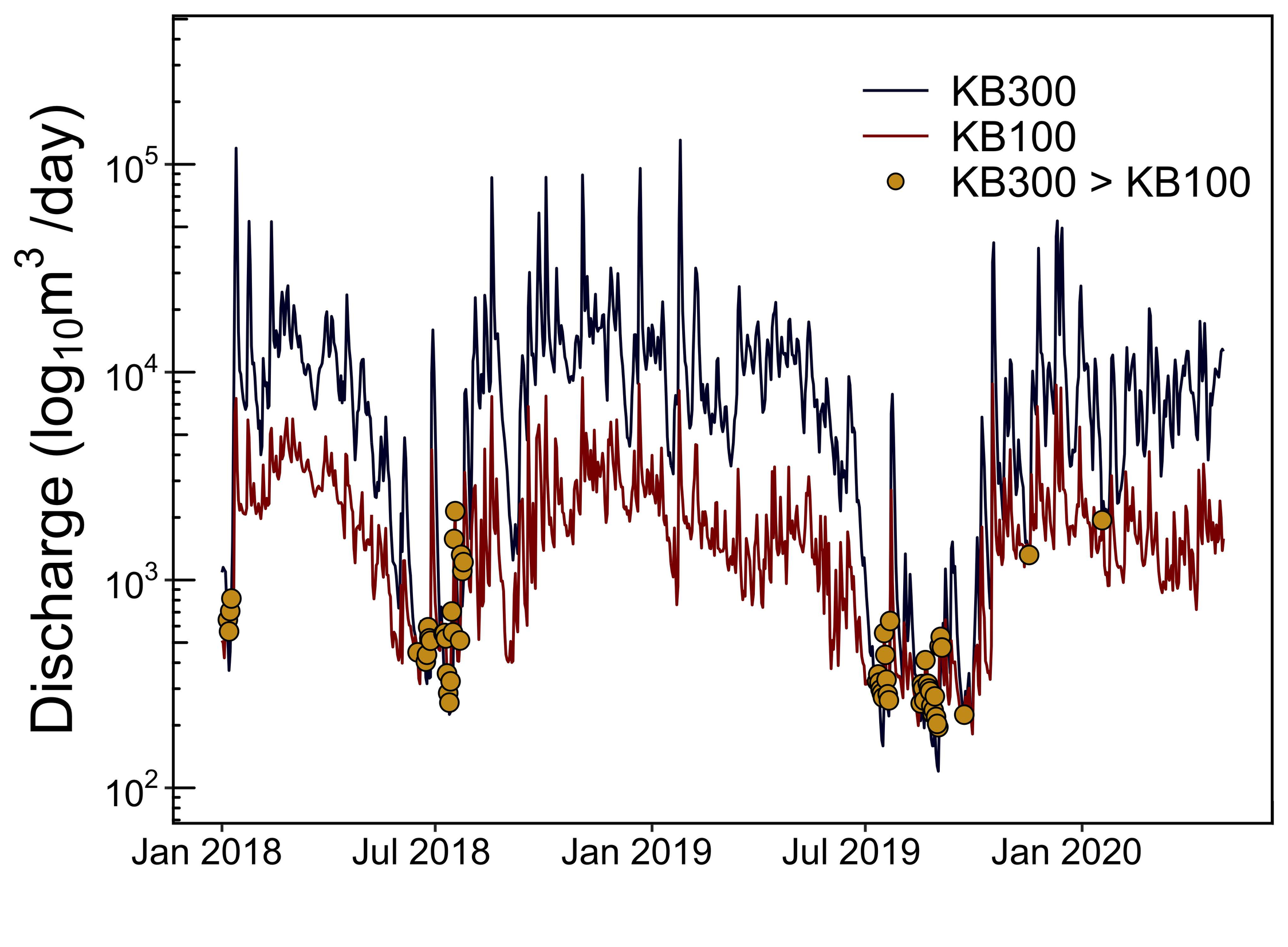

Supplement: S2 Fig — The grey dots indicate events when discharge is higher at the inlet during the dry months (June—Oct) during which Kampoosa Brook becomes a losing stream and recharges the fen region and groundwater. (TIF) [file pone.0312259.s002.tif]

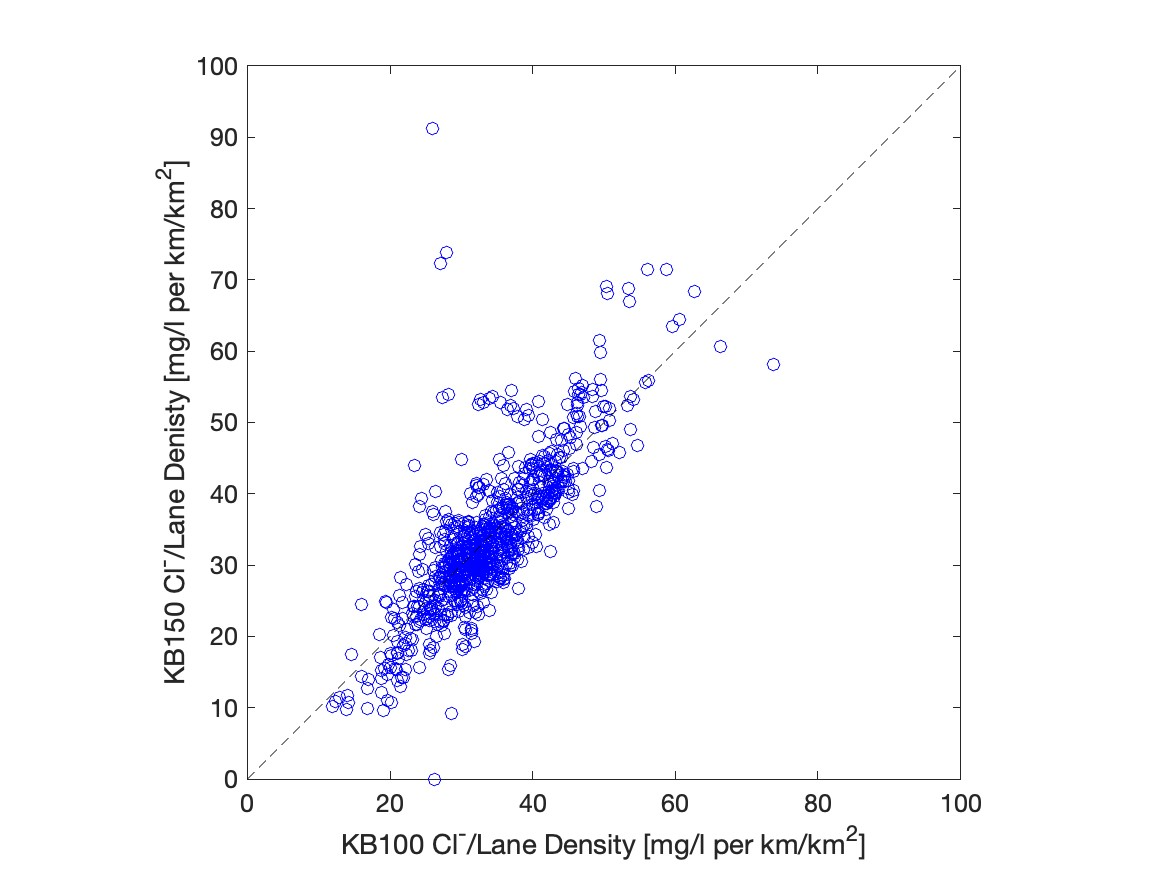

Supplement: S3 Fig — (TIF) [file pone.0312259.s003.tif]

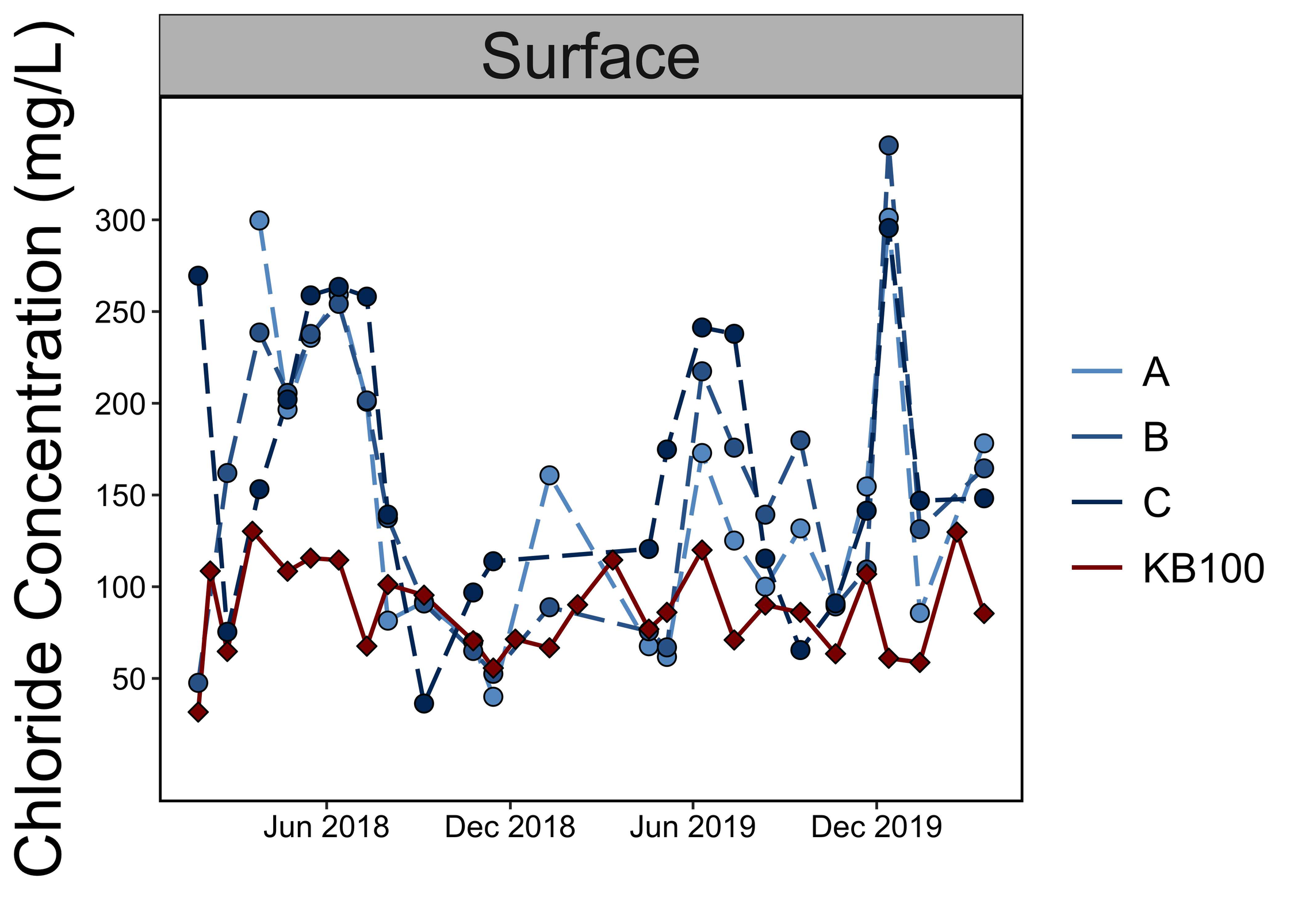

Supplement: S4 Fig — Surface water concentrations in the fen region tend to be higher. Unlike the groundwater chemistry at the 5ft and 15ft depths, surface water does not follow a distinct spatial pattern: concentrations are not always higher at well A which is closer to I-90. (TIF) [file pone.0312259.s004.tif]

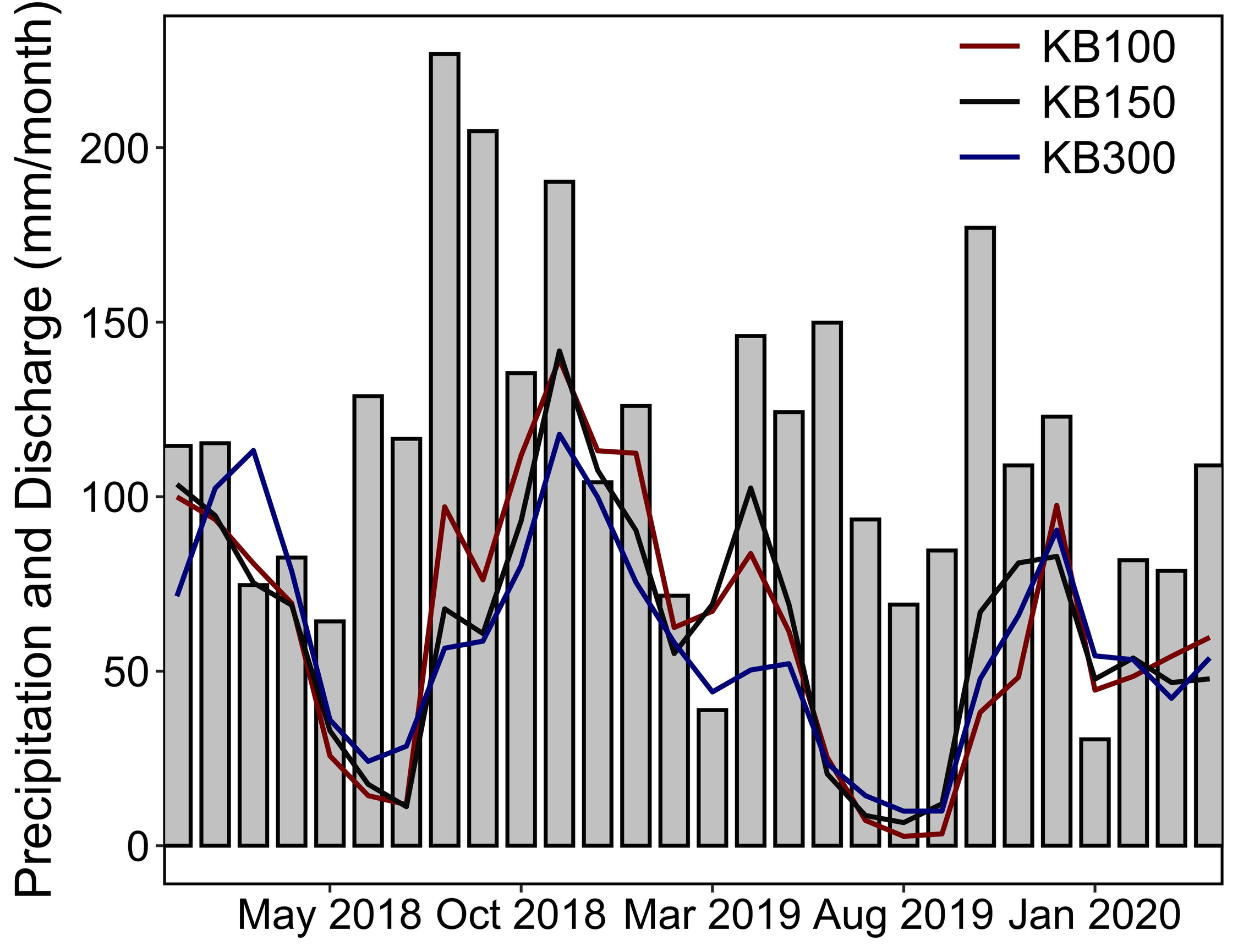

Supplement: S5 Fig — Precipitation is represented by the grey bars and the line graphs represent the different subwatersheds. High discharge is recorded during high precipitation events and in the Sept–Feb months. (TIF) [file pone.0312259.s005.tif]

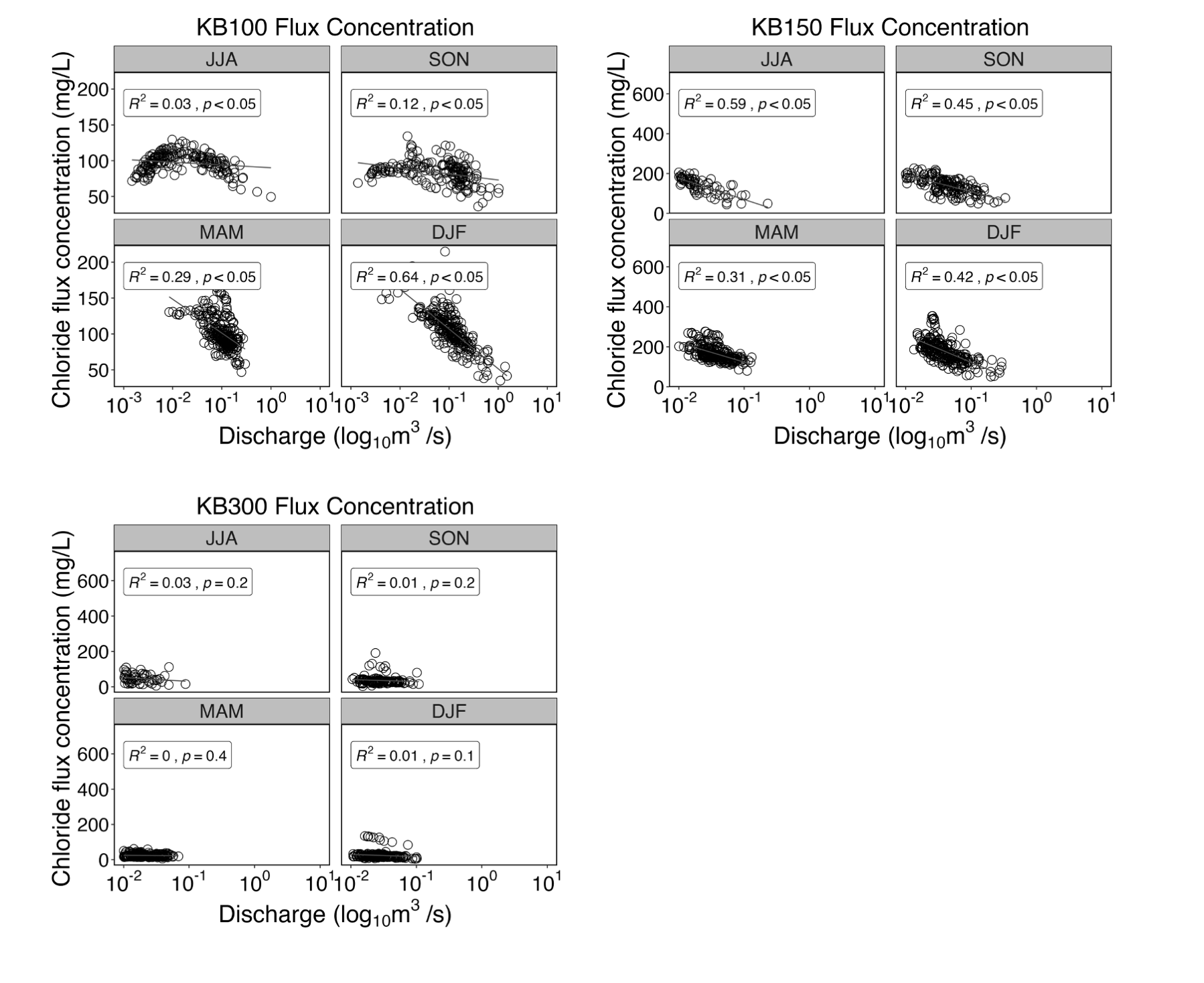

Supplement: S6 Fig — (TIF) [file pone.0312259.s006.tif]

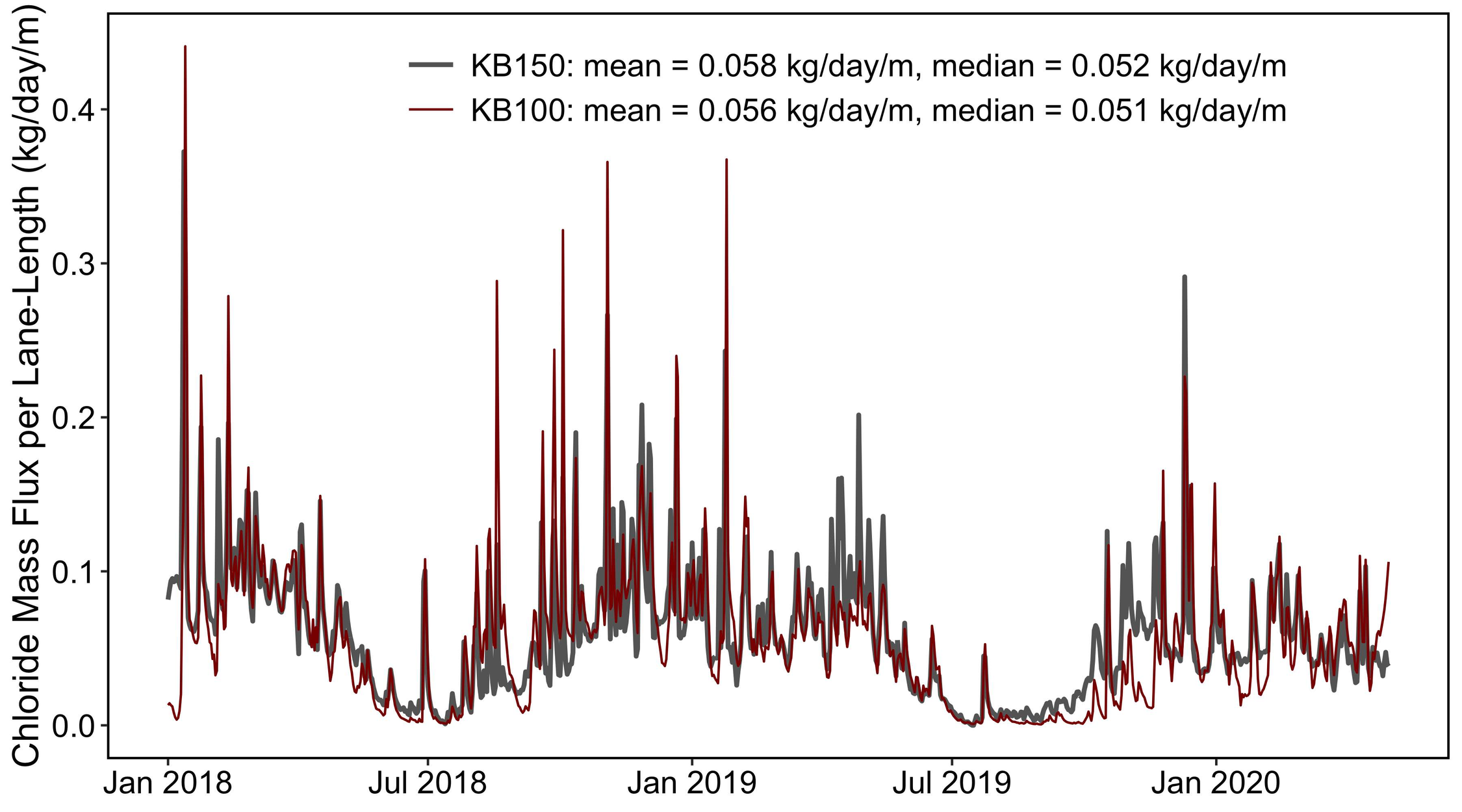

Supplement: S7 Fig — The mean and median mass fluxes are calculated. (TIF) [file pone.0312259.s007.tif]

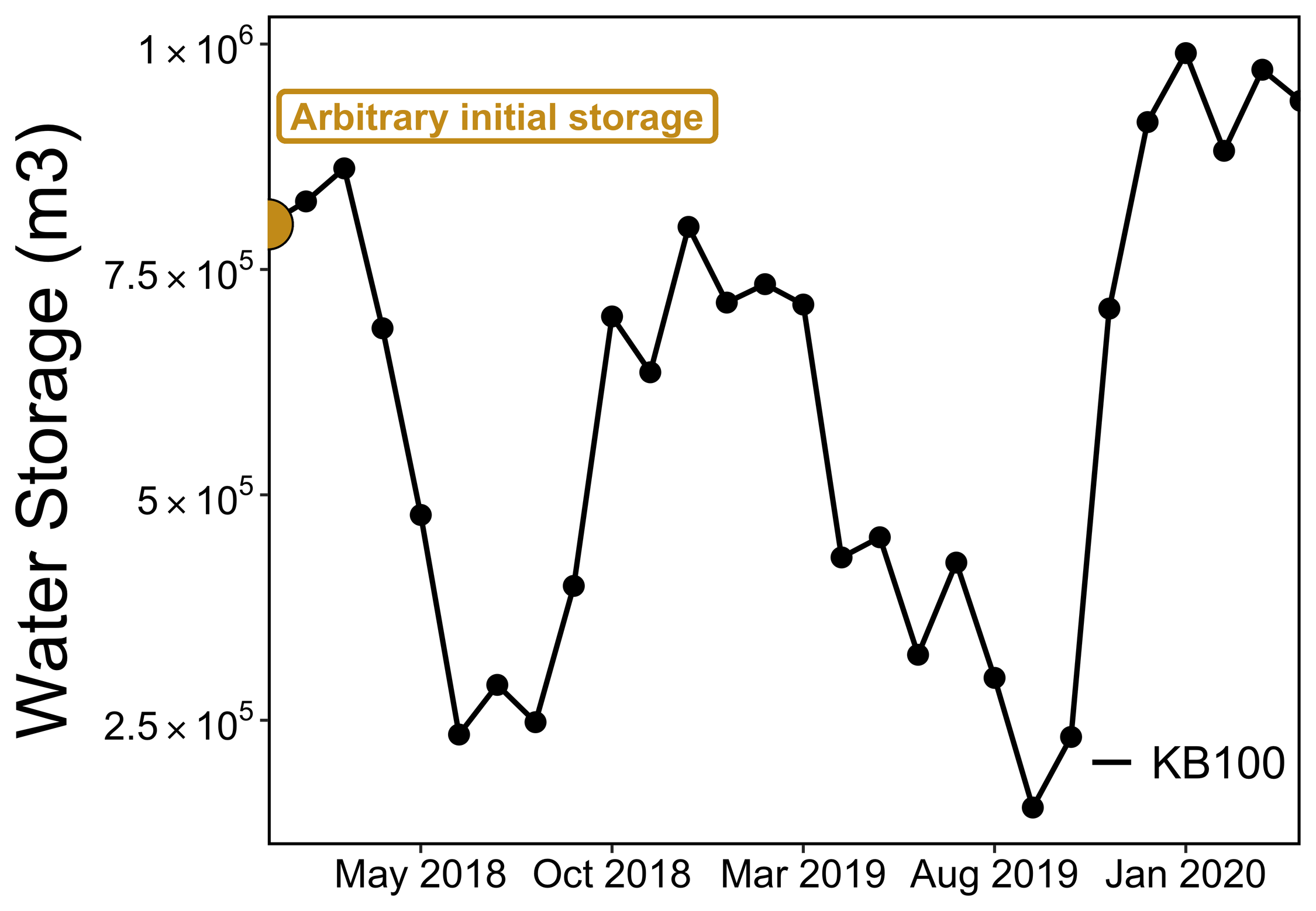

Supplement: S8 Fig — An arbitrary initial storage value of 800000 m3 was used in this study. Changes in water storage follow a seasonal pattern and water storage tends to be higher in the Sept—Feb months. (TIF) [file pone.0312259.s008.tif]

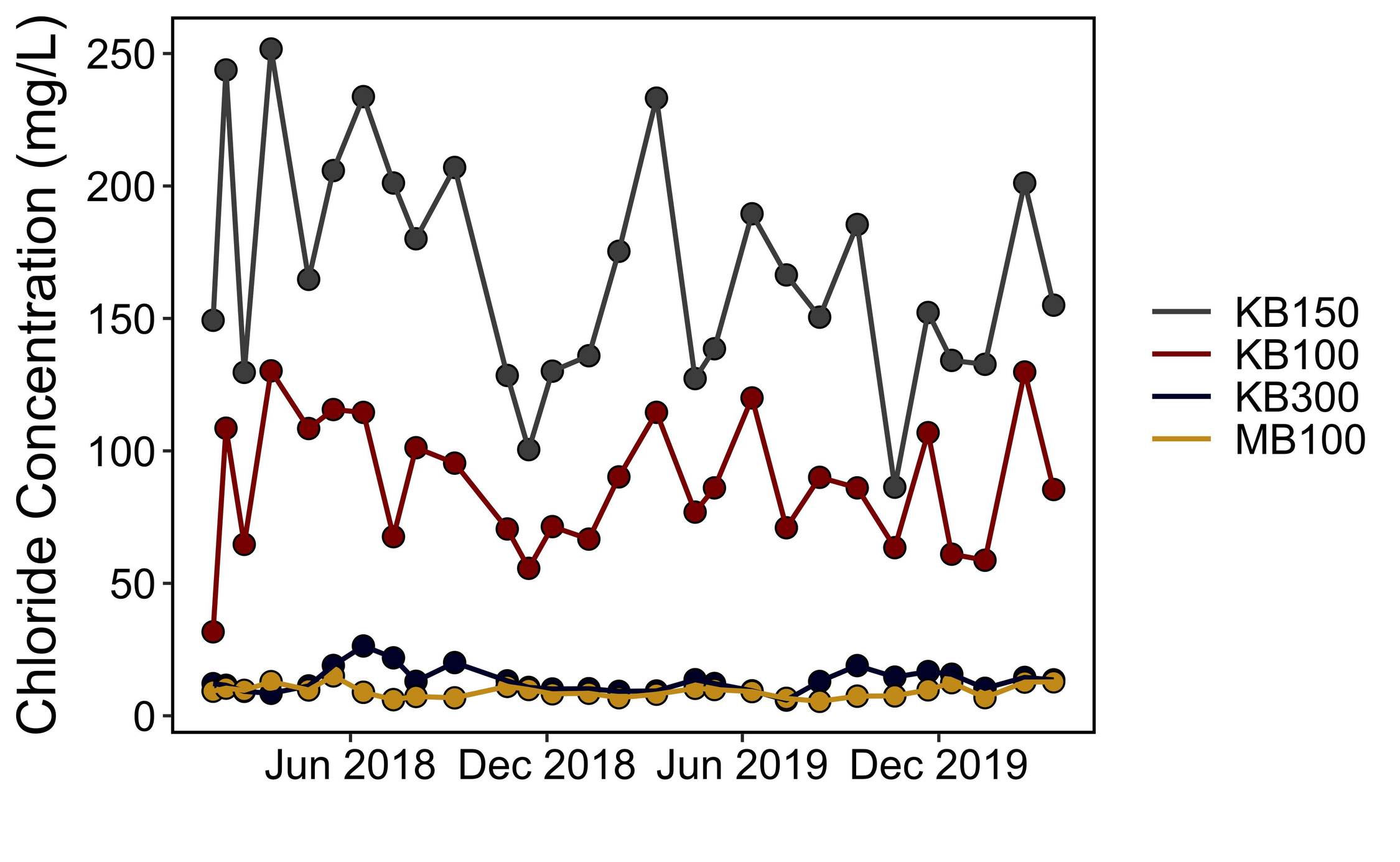

Supplement: S9 Fig — Concentrations at MB-100 are considered to be the background concentrations independent of road-salt added to I-90 and US-7. (TIF) [file pone.0312259.s009.tif]
