# Supplementary material for: Accumulation of road salt in a calcareous fen: Kampoosa Bog, western Massachusetts
Source: PLoS One. 2024 Oct 31;19(10):e0312259. doi: 10.1371/journal.pone.0312259 (PMC11527221; doi:10.1371/journal.pone.0312259)
Supplement: S1 Table — (DOCX) [file pone.0312259.s010.docx]

| **Year** | **Precipitation (mm)** | **Chloride Input**  **Wetland Region (kg)** | **Chloride Input KB150 (kg)** | **Chloride Input**  **KB300 (kg)** | **Chloride Input**  **KB100 (kg)** |
| --- | --- | --- | --- | --- | --- |
| 2012 | 1016 | 140000 | 169000 | 29000 | 338000 |
| 2013 | 880 | 120000 | 160000 | 25000 | 305000 |
| 2014 | 1171 | 166000 | 226000 | 34000 | 426000 |
| 2015 | 1097 | 133000 | 206000 | 27000 | 366000 |
| 2016 | 1153 | 138000 | 205000 | 28000 | 371000 |
| 2017 | 1002 | 149000 | 195000 | 30000 | 374000 |
| 2018 | 1552 | 194000 | 284000 | 40000 | 518000 |
| 2019 | 1334 | 161000 | 246000 | 33000 | 440000 |

**S1 Table**. Historic chloride application rates in the Kampoosa Bog subwatersheds between 2012

and 2019.
